# Meta-Analysis of Duplex Surveillance Following Lower Limb Endovascular Intervention

**DOI:** 10.1177/15266028231215215

**Published:** 2023-12-04

**Authors:** Mervyn McKenna, Hussein Elghazaly, Henry Bergman, Laura Wingate, Dan Robbins, Alun H. Davies, Ankur Thapar

**Affiliations:** 1 Academic Vascular Ultrasound Scientist and Research Fellow, Mid and South Essex Vascular Unit, Mid and South Essex NHS Foundation Trust, Biomechanics, Optics, Robotics and Imaging Group, Anglia Ruskin University, Cambridge, UK; 2 Academic Section of Vascular Surgery, Department of Surgery and Cancer, Imperial College London, London, UK; 3 Lead vascular ultrasound scientist, Mid and South Essex Vascular Unit, Mid and South Essex NHS Foundation Trust, Basildon, UK; 4 Biomechanics, Optics, Robotics and Imaging Group, Anglia Ruskin University, Cambridge, UK; 5 Circulatory Health Group, Anglia Ruskin University, Consultant Vascular and Endovascular Surgeon, Mid and South Essex NHS Foundation Trust, Basildon, UK

**Keywords:** peripheral arterial disease, duplex ultrasound, surveillance, endovascular therapy

## Abstract

**Introduction:**

The aim of this systematic review was to identify the evidence in the literature for limb salvage with the introduction of duplex surveillance.

**Methods:**

A systematic review and meta-analysis was performed using the Preferred Reporting Items for Systematic Reviews and Meta-Analysis guidelines (PRISMA) methodology for all studies which compared a group undergoing clinical surveillance with a group undergoing combined clinical and duplex surveillance after endovascular therapy for peripheral arterial disease. MEDLINE, EMBASE, the Cochrane Database for Systematic Reviews, and ClinicalTrials.gov were searched for relevant studies by 2 reviewers. Studies were quality assessed using the ROBINS-I tool. An individual patient data survival analysis and meta-analysis for 1- and 2-year amputation outcomes using a random-effects model were performed.

**Results:**

Two low-quality nonrandomized studies met the inclusion criteria. There was a statistically and clinically significant reduction in major amputation in patients undergoing combined clinical and duplex surveillance (log-rank p<0.001). The number needed to treat to prevent 1 amputation at 2 years was 5 patients. At 1 year, the odds ratio (OR) for amputation was 0.22, 95% confidence interval (CI)=0.10-0.48, with no statistical heterogeneity. At 2 years, the numbers of patients were low and the effect on amputation was less certain OR=0.25, 95% CI=0.04-1.58.

**Conclusions:**

Preliminary, low-quality data suggests that there may be a clinically significant reduction in major amputation with the introduction of duplex surveillance. It is recommended that a randomized controlled trial is performed to confirm these findings and identify the anatomical subgroups that benefit the most from surveillance.

**Clinical Impact:**

“Two low-quality studies reveal a significant clinical impact: combined clinical and duplex surveillance markedly reduces major amputations (log-rank p<0.001). At 1-year, the odds ratio for amputation is 0.22 (95% CI=0.10-0.48), emphasizing limb salvage benefits. Despite less certainty at 2-years, a notable absolute risk reduction of 19% is seen, with a number needed to treat of 5. This underscores the urgent need for a randomized controlled trial to validate findings and identify key subgroups. The meta-analysis strongly advocates implementing duplex surveillance for a year post-endovascular interventions, especially in patients fit for reintervention, with important considerations for cost-effectiveness and focused clinical trials.”

## Key Findings

There was a significant benefit for limb salvage in the duplex surveillance cohort (χ^2^=23; p<0.0001, log-rank). The absolute risk reduction at 2 years was 19%, with a number needed to treat of 5 to prevent 1 major lower limb amputation.

## Take Home Message

Preliminary, low-quality data suggest duplex is beneficial, but a randomized controlled trial is required to confirm these findings.

## Introduction

Endovascular therapy is now the most common mode of revascularization for peripheral arterial disease (PAD).^1^ In the 2017 to 2019 UK National Vascular Registry Report, 23 881 endovascular procedures were recorded in comparison to 18 090 surgical bypasses.^2^ When considering procedures performed for PAD, 39% of patients had intermittent claudication, 9% ischemic rest pain, and 43% ulceration or gangrene.^2^ These last 2 categories are classified as critical limb-threatening ischemia (CLTI)^3^—that is if blood flow is not restored, the result is major amputation or death.^3^

Endovascular therapy for chronic PAD encompasses balloon angioplasty, stenting, drug-coated balloon angioplasty, drug-eluting stents and atherectomy, alone or in combination.^3^ Endovascular therapy does not include thrombolysis or pharmacomechanical thrombectomy which are reserved for acute thromboembolism.^3^ In open bypass surgery, duplex ultrasound surveillance has not been shown to significantly reduce major amputation,^4^ when compared with clinical surveillance (consisting of a history of recurrence of ischemic pain, wound deterioration, pulse palpation, and bedside test such as ankle-brachial index, toe pressure, and Doppler waveforms).^4^

Recently, the European Society for Vascular Surgery (ESVS) and the European Society of Cardiology (ESC) published a joint recommendation for a 1-year duplex ultrasound surveillance program after endovascular therapy.^3^ The recommended surveillance consists of duplex imaging within 1 month (scan 1), within 6 months (scan 2), and at 12 months (scan 3).^3^ However, introduction of duplex surveillance may lead to further procedures, complications, hospital re-admissions, and costs. For example, at 1 UK vascular center, 800 extra duplex examinations per year were required for lower limb arterial surveillance,^5^ representing the additional workload for half a vascular sonographer, per year, per vascular unit.^5^ This represents a significant cost and workload to health care providers, in addition to more hospital visits for patients and the costs and risks of reintervention. The aim of this systematic review was to synthesize comparative studies of clinical surveillance vs combined clinical and duplex surveillance after endovascular therapy for lower limb PAD, to determine the evidence base for this practice.

## Methods

### Search Strategy and Selection Criteria

This systematic review and meta-analysis was conducted according the Preferred Reporting Items for Systematic Reviews and Meta-Analysis guidelines (PRISMA).^6^ MEDLINE, EMBASE, the Cochrane Database of Systematic Reviews, and *Clinicaltrials.gov* were searched for studies investigating the use of duplex ultrasonography in addition to clinical and hemodynamic parameters for the surveillance of patients with PAD after endovascular intervention. The full-search strategy can be found in the Supplementary Material (S1). Additional literature was identified by cross-checking the reference lists and citing articles of eligible studies (including the European Guideline)^3^. There were no restrictions on publication language, date, or status, and the search was last updated on August 10, 2022.

### Inclusion and Exclusion Criteria

Inclusion criteria were 2 arm studies comparing amputation outcomes in adults after clinical vs combined clinical and duplex surveillance after lower limb endovascular therapy for PAD. Exclusion criteria were review articles, letters, single arm studies with no comparator, and studies with less than 1 year of follow-up.

### Data Extraction

Search results were imported into Covidence^7^ for abstract screening; duplicates and irrelevant studies were removed, based on the above eligibility criteria. Two independent authors (M.M. and H.E.) reviewed potential studies. Full texts of studies were subsequently retrieved and reviewed against the same criteria. Discrepancies were adjudicated by a third, experienced author (A.T.). When the data from eligible studies were insufficient, the authors were contacted.

### Assessment of the Quality of Included Studies

The ROBINS-I tool was used for quality assessment of the nonrandomized studies found, as recommended by Cochrane.^8^

### Endpoints

The primary endpoint was limb salvage. The secondary endpoints were mortality and primary-assisted patency. The endpoints were selected as they reflect successful reintervention based on ultrasound findings to prevent re-occlusion—the mechanism for duplex surveillance reducing major amputation. Mortality was chosen, as it reflects the appropriateness of patients for further intervention and reflects the effect of serious complications incurred during revision procedures.

### Statistical Analysis

Quantitative synthesis of included studies was performed in Review Manager 5.4 (RevMan, Cochrane, London). Odds ratios (ORs) for each were pooled with the random-effects model using the Mantel-Haenszel test in RevMan. Where data were noted to be insufficient for meta-analysis, the results were presented qualitatively.

### Individual Patient Data Meta-Analysis

Individual patient data were extracted from individual study Kaplan-Meier survival curves. The method for extraction of individual patient data has been previously described.^9,10^ Briefly, a web-based tool^11^ was used to digitize individual Kaplan-Meier data. Iterative numerical methods combined digitized Kaplan-Meier data numbers of patients at risk (included in tables below published Kaplan-Meier curves) to reconstruct individual patient data. Analyses were performed using R Version 4.0.5 and RStudio 2022.07.1+554.^12^

Using the “survival,” “ggsurvfit,” and “survimer” packages in R, individual patient data from individual studies were combined to produce a Kaplan-Meier survival curve for each surveillance arm. Numbers at risk, cumulative numbers of events, and cumulative numbers of patients censored were reported. A log-rank test was used to compare freedom from the primary endpoint between the arms. Statistical significance was set at p<0.05.

## Results

### Database Search Results

**Figure 1.**
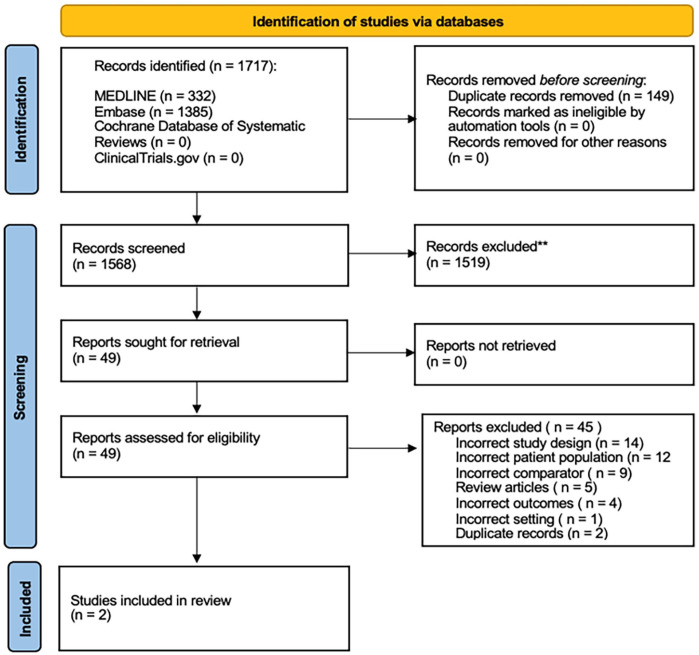
PRISMA flowchart.

Figure 1 shows the PRISMA diagram.

### Study and Baseline Characteristics

The characteristics of the 2 included studies are summarized in Table 1. Briefly, Draxler et al^13^ was a retrospective, nonrandomized single-center study in which 248 patients were treated with a superficial femoral artery (SFA) stent. The authors investigated whether the additional use of duplex stent imaging improved outcomes compared with clinical and bedside Ankle Brachial Pressure Index (ABPI) surveillance. The average follow-up was 36 months.

**Table 1.** Study Characteristics.

Author	Draxler et al	Martinez-Rico et al
Year of publication	2021	2022
Number of patients	248	305
Number of centers	1	1
Study design	Retrospective, nonrandomized	Prospective, nonrandomized
Average length of follow-up	36 months	18 months

**Figure 2.**
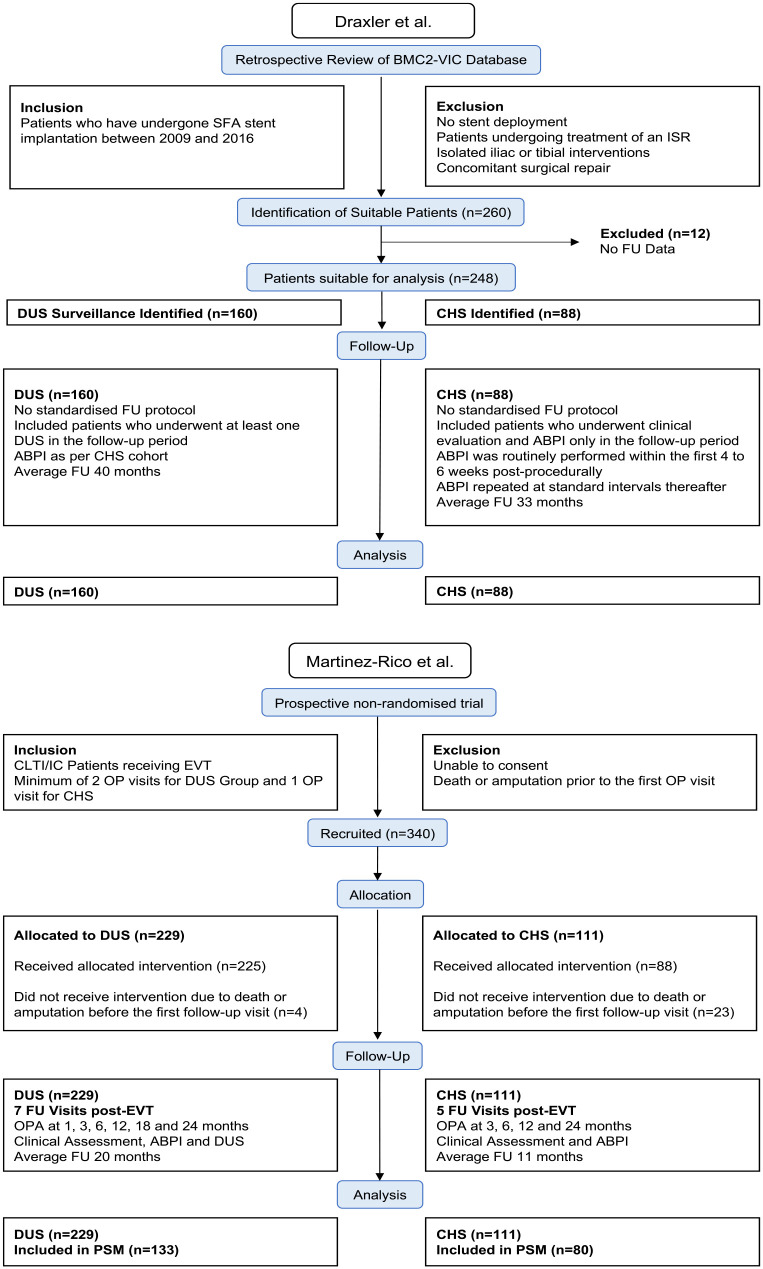
Protocols of included studies. ABPI, Ankle Brachial Pressure Index; CHS, clinical and hemodynamic surveillance; CLTI, critical limb-threatening ischemia; DUS, duplex ultrasound surveillance; EVT, endovascular therapy; FU, follow-up; IC; intermittent claudication; SFA; superficial femoral artery.

The study by Martinez-Rico et al^14^ was a prospective, nonrandomized single-center study in which 305 patients with suprainguinal or infrainguinal disease were treated. The authors investigated whether the use of duplex improved outcomes compared to clinical and bedside ABI surveillance. Average follow-up was 18 months (11 months for the clinical surveillance cohort and 20 months for the combined clinical and duplex surveillance cohort). A summary of the protocols is provided in Figure 2.

### Heterogeneity

Baseline patient and lesion characteristics from both studies are summarized in Table 2. Of note, in Draxler et al,^13^ the clinical surveillance group had a higher incidence of ischemic heart disease, end-stage renal disease, and represented a population with greater comorbidity. In Martinez-Rico et al,^14^ diabetes was more common in the combined duplex and clinical surveillance group. There was a higher proportion of female patients in the Draxler et al^13^ study when compared with Martinez-Rico et al.^14^ Furthermore, the proportion of patients with CLTI was 60% in Draxler et al^13^ compared with 80% in Martinez-Rico et al.^14^

**Table 2.** Baseline Demographics.

Author	Draxler et al		Martinez-Rico et al	
Surveillance type	Duplex	Clinical	Duplex	Clinical
Average age (yrs)	69	68	68	70
Smokers (%)	73	68	NA	NA
Female (%)	45	45	26	26
Diabetes (%)	53	59	72	55
Hypertension (%)	94	99	88	83
Dyslipidemia (%)	85	86	72	73
Ischemic heart disease (%)	38	52	44	40
Dialysis (%)	5	13	NA	NA
Cancer (%)	NA	NA	NA	NA
Lesion location (%)				
Iliac	0	0	33	42
Femoropopliteal	100	100	52	44
Tibial	0	0	16	14
Lesion/stent length (mm)	160	140	NA	NA
TASC class (%)				
A	21	17		
B	43	45	NA	NA
C	16	16		
D	20	22		
Clinical presentation (%)				
Claudication	41	38	17	26
Rest pain	18	19	20	15
Tissue loss	41	43	63	59
Stent deployment	100%	100%	36%	32%
Type of stent (%)				
BMS	94	96	NA	NA
DES	0	2	NA	NA

Abbreviation: NA, not available, TASC, TransAtlantic Inter-Society Consensus, BMS, Bare metal stent, DES, Drug eluting stent.

Several differences in the methodologies and study protocols exist. These are summarized in Supplementary Table S2 in the Supplementary Material. For instance, whereas Draxler et al^13^ only included patients who received stents for treatment of SFA lesions, Martinez-Rico et al^16^ included suprainguinal and infrainguinal disease and patients undergoing angioplasty alone. In addition, as opposed to Draxler et al,^13^ Martinez-Rico et al^14^ allowed inclusion of patients undergoing reintervention as well as those who had concomitant femoral endarterectomy. Furthermore, differences were noted in the protocol for postprocedural antiplatelet therapy and the definitions of re-stenosis, thus inferring differences in the parameters utilized to guide reintervention.

### Quality of Included Studies

**Figure 3.**
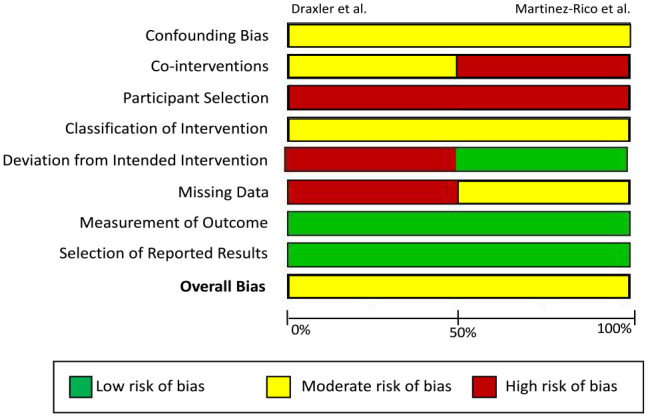
Summary of risk of bias in the study. All bias domains were deemed as “Low Risk,” “Moderate Risk,” or “High Risk” according to the ROBINS-I tool. Confounding bias refers to bias introduced due to imbalances of confounding variables including demographics, comorbidities, and lesion/procedural characteristics. Bias due to cointerventions includes variations in concomitant surgical/hybrid procedures as well as best medical therapy. Bias due to participant selection denotes allocation bias that is introduced when assigning participants to their respective study arms. Bias due to classification of intervention encompasses the definition of interventions received by participants and whether intervention status label could have been affected by knowledge of the outcome. Deviation from intended intervention denotes nonconformity of interventions received by patients to the study’s original protocol. Missing data investigate bias of outcomes due to unbalanced follow-up in each study arm and imbalances in patients lost to follow-up. Measurement of outcome investigates whether knowledge of the intervention received may have influenced the outcome. Selection of reported results investigates bias investigates whether authors have reported results in a manner that biases the findings of the study. This includes reporting multiple outcome measurements within the outcome domain, reporting multiple analyses of the intervention-outcome relationship, and reporting different subgroups.

Overall, there was a moderate risk of bias in both studies (Figure 3). Confounding bias was evident as both studies were nonrandomized, and there was evidence of differing proportions of known (renal failure, ischemic heart disease) and unknown confounders between the arms, such as antiplatelet and statin therapy. Notably, both studies had significant differences in all-cause mortality between both cohorts, suggesting less fit patients may have preferentially entered the clinical surveillance arm; as such, both studies were at high risk of allocation bias in the “participant selection” domain. Antiplatelet therapy was not standardized, and Martinez-Rico et al^14^ included patients with hybrid procedures and were at risk of bias due to cointerventions. Draxler et al^13^ had no prespecified protocol, and hence, they had disparity in the duration of follow-up between arms and in attrition of participants. As such, Draxler et al^13^ was identified to have a high risk of bias in “deviation from intended intervention domain” and the “missing data” domain, respectively. As major lower limb amputation is an objective endpoint which does not require blinding, it was deemed a low risk of bias in the “measurement of outcome” domain for both studies.

### Primary Outcome: Incidence of Major Amputation

Both included studies reported the rates of major amputation at 12 and 24 months. Data are presented in Table 3. At 12 months, both studies found a significant benefit for duplex surveillance. Meta-analysis demonstrated amputations occurred in 10/275 patients in the duplex ultrasound surveillance group compared to 18/120 in the clinical and hemodynamic group (OR=0.22, 95% confidence interval [CI]=0.10-0.48) with no statistical heterogeneity (I^2^=0%).

**Table 3.** Absolute Rates of Lower Limb Amputation at 12, 18, 24 and 36 Months.

	Major lower limb amputations							
Author	Draxler et al				Martinez-Rico et al (PSM)			
Surveillance Type	Duplex		Clinical		Duplex		Clinical	
	Events	Total	Events	Total	Events	Total	Events	Total
12 months	5	150	11	65	5	125	7	55
18 months	6	122	17	51	8	121	7	45
24 months	7	117	17	44	10	93	8	51
36 months	9	90	18	36	10	41	8	16

Abbreviation: PMS, Propensity Score Matched.

At 24 months, Draxler et al^13^ found a significant benefit for duplex ultrasound surveillance, whereas Martinez-Rico et al^14^ found no difference in this outcome. Meta-analysis demonstrated amputations occurred in 17/210 patients in the duplex ultrasound surveillance group compared with 25/95 in the clinical and hemodynamic group (OR=0.25, 95% CI=0.04-1.58). There was significant statistical heterogeneity between the studies (I^2^=85%). Supplemental Figure S3 in the Supplementary Material shows the forest plots of this analysis.

**Figure 4.**
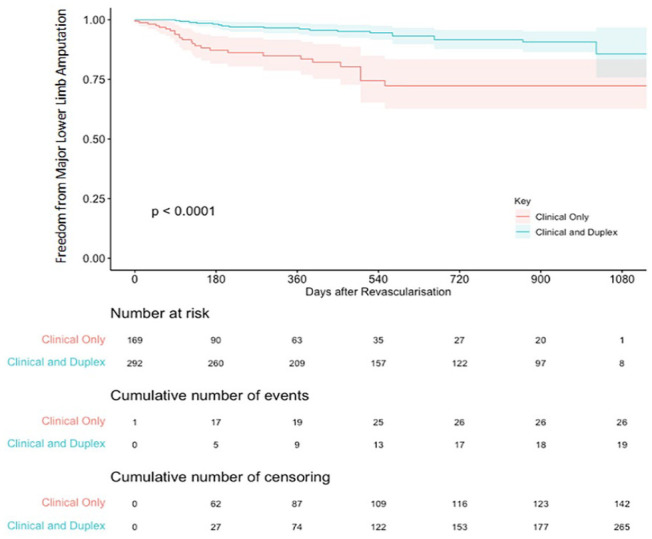
Kaplan-Meier survival curve for freedom from major lower limb amputation. The curves depict pooled results for the primary endpoint from the 2 available studies.

A Kaplan-Meier survival curve of the individual patient data is shown in Figure 4. There was a significant benefit for in freedom from major lower limb amputation in the duplex surveillance cohort compared with the clinical and hemodynamic surveillance cohort (χ^2^=23; p<0.0001, log-rank). The absolute risk reduction at 2 years was 19%, with a number needed to treat (NNT) of 5 to prevent 1 major lower limb amputation.

### Secondary Outcomes

Data were insufficient to perform meta-analysis for our secondary outcomes. Results are summarized in Table 4.

**Table 4.** Absolute Rates of Primary-Assisted Patency and All-Cause Mortality at 12, 24 and 36 Months.

	Primary-assisted patency (Draxler et al)				All-cause mortality (Martinez-Rico et al)			
	Duplex		Clinical		Duplex		Clinical	
	Events	Total	Events	Total	Events	Total	Events	Total
12 months	110	131	48	63	9	110	15	59
24 months	84	112	23	45	13	91	17	48
36 months	66	97	15	39	14	85	18	30

### Primary-Assisted Patency

Draxler et al^13^ reported 12-, 24-, and 36-month rates of primary-assisted patency of 84%, 75%, and 68%, respectively, in combined clinical and duplex surveillance arm vs 76%, 51%, and 38% in the clinical surveillance cohort.^13^ Martinez-Rico et al^14^ did not report data on primary-assisted patency.

### All-Cause Mortality

Martinez-Rico et al^14^ reported 12-, 24-, and 36-month mortality rates of 8%, 14%, and 16%, respectively, in combined clinical and duplex surveillance arm vs 25%, 35%, and 60% in the clinical surveillance cohort. Martinez-Rico et al^14^ performed a log-rank test of their Kaplan-Meier plot, which showed a significant difference between both cohorts (p<0.001). Although Draxler et al^13^ did not report mortality throughout follow-up, the authors report that survival in the duplex surveillance group was higher, with 90% of the patients still alive after 60 months compared with 50% of patients followed up by clinical surveillance alone (p=0.017).

### Publication Bias

Publication bias was not examined quantitatively due to the low number of studies; however, both studies reported beneficial outcomes for duplex surveillance.

## Discussion

This is the first systematic review to examine the benefits of combined clinical and duplex surveillance after lower limb endovascular therapy. The main finding was a significant benefit for duplex surveillance at preventing amputations, although greater confidence can be associated with the results at the 1-year timepoint due to the reduced number of patients at risk after this time period. The increased confidence at the 1-year timepoint was apparent both for superficial femoral artery stents and lower limb angioplasty with provisional stenting in general. The number of patients undergoing surveillance to prevent 1 amputation was only 5 patients, indicating a possibly large and clinically relevant effect.

It has been suggested that improved clinical outcomes of duplex surveillance are driven by better primary-assisted patency,^16–19^ ie, successful reintervention to prevent thrombosis.^16–19^ However, at 1 year, primary patency was only marginally better, but it had diverged at 2 years. Other explanations, such as differences in antithrombotic therapy, wound depth, osteomyelitis, or foot deformity in the duplex surveillance arms are possible, as well as the risks of unknown confounders. In included studies, mortality was very high in the clinical surveillance arm, resulting in very small numbers at risk after 18 months, and hence unreliable conclusions from then on.

The strengths of this study were that it used a robust methodology to identify comparative studies where amputation rates could be analyzed in 2 separate arms, rather than a single cohort which underwent both surveillance strategies. Data were quantitatively analyzed and numbers were adequate to assess early outcomes. Major amputation rates were chosen as a hard endpoint not prone to observer bias and relevant to the study populations, which were mainly CLTI patients.

The limitations of this study were that there was a substantial risk of bias from selection of fitter patients into the intensive surveillance and reintervention arms, as well as other confounders which may have biased the results in favor of the duplex surveillance arm. There were no parameters given for reintervention and complications were poorly described. Importantly outcomes of those patients undergoing salvage procedures were not described. Medical therapy was not standardized, and some patients had co-interventions such as endarterectomy or a second antiplatelet agent. Finally, this analysis reflects data from the 2 small, low-quality, comparative studies that are currently available in the literature.

For comparison, the 2018 Society for Vascular Surgery recommendations were made on the basis of Level C evidence. These were for aorto-iliac intervention combined clinical, hemodynamic and duplex surveillance at 1, 6, and 12 months, followed by annual clinical and hemodynamic examination. For femoropopliteal intervention combined clinical, hemodynamic and duplex surveillance at 1, 3 and 6 months, followed by every 6 months for patients with stents or with CLTI. For tibial intervention combined clinical, hemodynamic, and duplex surveillance at 1 month, followed by clinical surveillance alone at 3 and 6 months. In contrast, the UK Provision of Vascular Services document does not even mention surveillance after intervention.^19^ Although surveillance based on segment treated may seem sensible, 47% of patients treated in the UK 2021 National Vascular Registry had multiple vessels treated simultaneously for CLTI. These patients have the most to lose from re-occlusion and hence were the focus of this study.

Finally the introduction of duplex surveillance actually represents a strategy of reintervention to improve primary-assisted patency in fitter patients. It is important that a trial be undertaken, because data from bypass versus angioplasty for severe ischaemia of the limb (BASIL) demonstrates that salvage bypass after failed endovascular therapy has a 20% worse amputation-free survival than primary bypass, an 8% mortality, and a 39% morbidity.^20^ We should not subject patients to more high-risk reintervention, without evidence that it prevents amputation, improves quality of life, independence in daily activities, and pain levels. We currently have little information on the latter 3, which are vital patient centered outcomes.

## Conclusions

The findings of this meta-analysis lend support to the ESVS/ESC recommendation^4^ to introduce duplex surveillance after endovascular interventions for 1 year, in patients who are fit enough for reintervention. It would appear that in patients unfit for reintervention, clinical surveillance remains sufficient. Although Society for Vascular Surgery (SVS) recommendations are published for each anatomical territory, many patients with CLTI undergo treatment of multiple arterial territories at the same time. Undertaking a randomized control trial for this population would provide robust evidence to support surveillance or not and to examine the anatomical subgroups that might benefit the most. Another consideration is the cost-effectiveness of ultrasound surveillance and workforce requirements and cost and complications of reinterventions to health care providers. The outcomes of those undergoing salvage procedures also require better characterization. It is recommended that a randomized controlled trial of clinical vs combined clinical and duplex surveillance in patients with CLTI is now performed.

## Supplemental Material

sj-docx-1-jet-10.1177_15266028231215215 – Supplemental material for Meta-Analysis of Duplex Surveillance Following Lower Limb Endovascular Intervention Supplemental material, sj-docx-1-jet-10.1177_15266028231215215 for Meta-Analysis of Duplex Surveillance Following Lower Limb Endovascular Intervention by Mervyn McKenna, Hussein Elghazaly, Henry Bergman, Laura Wingate, Dan Robbins, Alun H. Davies and Ankur Thapar in Journal of Endovascular Therapy
