# Supplementary material for: Meta-Analysis of Duplex Surveillance Following Lower Limb Endovascular Intervention
Source: J Endovasc Ther. 2023 Dec 4;32(5):1351–9. doi: 10.1177/15266028231215215 (PMC12433530; doi:10.1177/15266028231215215)
Supplement: sj-docx-1-jet-10.1177_15266028231215215 – Supplemental material for Meta-Analysis of Duplex Surveillance Following Lower Limb Endovascular Intervention [file sj-docx-1-jet-10.1177_15266028231215215.docx]

**Supplementary Material**

**Supplementary Material (S1): Search Strategy**

1 Ultraso*.m_titl. (391589)

2 Doppler.m_titl. (78734)

3 Duplex.m_titl. (18215)

4 Triplex.m_titl. (2881)

5 Non-invasive.m_titl. (48573)

6 1 or 2 or 3 or 4 or 5 (513643)

7 endovascular.m_titl. (68708)

8 angioplasty.m_titl. (43488)

9 atherectomy.m_titl. (4870)

10 stent.mp. [mp=ti, ab, hw, tn, ot, dm, mf, dv, kf, fx, dq, bt, nm, ox, px, rx, an, ui, sy] (310220)

11 7 or 8 or 9 or 10 (389698)

12 surveillance.m_titl. (114699)

13 follow-up.m_titl. (268609)

14 12 or 13 (382260)

15 6 and 11 and 14 (518)

16 remove duplicates from 15 (332)

| **Supplementary Table S2: Key Differences in the methodologies and protocols of the included studies** | | |
| --- | --- | --- |
|  | **Draxler et al.** | **Martinez-Rico et al.** |
| **Inclusion and Exclusion Criteria** | The authors included all patients who have undergone SFA stent implantation between 2009 and 2016. The patients included both those with intermittent claudication as well as critical limb ischaemia.  Patients were excluded if no stent was deployed during an endovascular procedure; if lesions were not de-novo/ if they were undergoing re-intervention for an in-stent restenosis (ISR); if the intervention did not involve stenting to the SFA, such as isolated iliac or tibial interventions; and if they received concomitant surgical repair along with their procedure. | The authors also included CLTI/IC Patients receiving EVT; however, unlike Draxler et al., patients were not excluded if they did not receive a stent.  Unlike Draxler et al., the authors included all treatment of multiple territories of the lower limb arteries, including iliac, femoropopliteal and BTK. Authors also allowed inclusion of patients undergoing re-intervention for an ISR as well as those who have had concomitant surgical repair along with their procedure. |
| **Post-Procedural APT** | All patients had postinterventional dual antiplatelet therapy with aspirin 81 mg/d (lifelong) and clopidogrel 75 mg/d (3-6 months) when no contraindication existed. As such, DAPT duration was shorter compared to the patients in Martinez-Rico et al. | Antiplatelet medication (aspirin or clopidogrel) was not discontinued before EVT.  If not already initiated, dual antiplatelet therapy was prescribed for 12 months, except if an increased risk of bleeding were suspected (cancer, peptic ulcer disease, renal impairment, anaemia, bleeding history or alcoholism).  In those cases, dual antiplatelet therapy was given for one to six months, depending on the lesion’s location and the surgeon's discretion.  Aspirin (monotherapy) was prescribed for 12 months for patients already on warfarin, and statins were routinely used in all patients. |
| **Clinical and Haemodynamic Follow-Up Protocol** | As this was a retrospective study, there was no standardised follow-up protocol in patients receiving clinical and. haemodynamic surveillance. This arm of the study included patients who did not undergo any duplex ultrasound scans in the follow-up period. ABPI was routinely performed within the first 4 to 6 weeks post-procedurally and was repeated at standard intervals thereafter.  The averave length of follow-up in this cohort was 33 months. The authors report an average of 2 follow-up examinations for this cohort of patents. This represents a much less frequent surveillance rate compared to Martinez Rico et al (2 visits per 33 months compared to 5 visits per 24 months). | There was a standardised protocol for patients who were allocated to the clinical and haemodynamic surveillance cohort. This included five outpatient follow-up visits post-procedurally. These outpatient appointments were at 3, 6, 12 and 24 months after endovascular treatment. All patients were followed-up for 24 months. Clinical Assessment and ABPI were performed in each visit. |
| **Duplex Ultrasound Surveillance Follow-Up Protocol** | As this was a retrospective study, there was no standardised follow-up protocol in patients receiving the duplex ultrasound surveillance. This arm of the study included patients who underwent at least one duplex ultrasound scan in the follow-up period.  The averave length of follow-up in this cohort was 41 months. The authors report an average of 3 follow-up examinations for this cohort of patents; however, they do not provide further insight inro whether duplex ultrasound was performed in each visit. Even if we assume all visits involved a duplex ultrasound scan, this represents a much less frequent surveillance rate compared to Martinez Rico et al (3visits per 41 months compared to 7 visits per 24 months).  The average time from stent insertion to the first stent imaging was 52 days, which is almost double the time for first duplex surveillance in Martinez-Rico et al. | There was a standardised protocol for patients who were allocated to the duplex ultrasound surveillance cohort. This included seven outpatient follow-up visits post-procedurally. These outpatient appointments were at 1, 3, 6, 12, 18 and 24 months after endovascular treatment. All patients were followed-up for 24 months.  Clinical Assessment and ABPI were conducted routinely at 3, 6, 12 and 24 months. |
| **Duplex Ultrasound Surveillance and Parameters** | The peak systolic velocity (PSV) was obtained at the following reference points in the vessel: common femoral artery, 3 cm proximal to where the stent starts, proximal third of the stent, mid stent, distal third of the stent, and suprageniculate popliteal artery. A ratio of PSVs, the PSV ratio (PSVR), was obtained at all segments within the stent comparing the obtained PSV with the reference PSV of the SFA proximal to the stent. In case of flush SFA stent location, the velocity of the common femoral artery was used as the refence point. **A PSV >250 cm/s or a PSVR >2.5 was regarded as a stenosis.** | Authors describe that DUS were performed by either a vascular surgeon or a certified vascular sonography technologist, highly experience in vascular ultrasound using the same US system (EPIQ S, (Philips, Eindhoven, the Netherlands). A focused DUS examination of the endovascular treated segment was performed, with a special focus on significant haemodynamic lesions defined by **a peak systolic velocity (PSV) > 300cm/s or PSV velocity ratio >3, corresponding to a 70% diameter reducing stenosis.**  As such, different cut-off values were used to define stenosis and guide intervention between both studies. |
| **Reintervention** | As this was a retrospective study, there was no standardised protocol to guide reintervention. The authors maintain that the decision to intervene on a detected stenosis was primarily determined by the vascular specialist and was based on the significance or progression of the stenosis, the patient’s overall clinical status, and the risk of reintervention. | Authors defined reintervention as a repeated treatment of stenosis/occlusions of the initial EV treated segment. Indication for reintervention was different between the two protocols.  In the duplex ultrasound-based surveillance protocol, reintervention was indicated by either deterioration in clinical status, an ABPI drop > 15 mmHg, or DUS-verified significant lesions with an unchanged or deterioration of clinical status irrespective of ABPI.  In the clinical and haemodynamic surveillance protocol, reintervention was driven only by deterioration in clinical status and/or ABPI. |

**Supplementary Figure S3: Forest Plots for Meta-analysis of Major Lower Limb Amputations**
